# Editorial: Application of Optimization Algorithms in Chemistry

**DOI:** 10.3389/fchem.2020.00198

**Published:** 2020-03-20

**Authors:** Jorge M. C. Marques, Emilio Martínez-Núñez, William L. Hase

**Affiliations:** ^1^Coimbra Chemistry Centre (CQC), Department of Chemistry, University of Coimbra, Coimbra, Portugal; ^2^Departmento de Química Física, Facultade de Química, Universidade de Santiago de Compostela, Santiago de Compostela, Spain; ^3^Department of Chemistry and Biochemistry, Texas Tech University, Lubbock, TX, United States

**Keywords:** molecular geometry search, fitting potential energy functions, spectral assignment, global optimization algorithms, energy landscapes

Molecular structure optimization, fitting potential energy functions to *ab initio* and experimental data, and spectral assignment are among the hardest optimization tasks in molecular sciences. These are fundamental problems in chemistry, but they can also be relevant in molecular physics and biochemistry. In past decades, several methodologies have been proposed to help in the above mentioned tasks, and some of them are already incorporated into computational tools, such as GMIN (Wales and Scheraga, 1999; Wales, 2010), Gradient Embedded Genetic Algorithm or GEGA (Alexandrova and Boldyrev, 2005), OGOLEM (Hartke, 1993; Dieterich and Hartke, 2017), Birmingham Cluster Genetic Algorithm or BCGA (Johnston, 2003; Shayeghi et al., 2015), Evolutionary Algorithm for Molecular Clusters or EA_MOL (Llanio-Trujillo et al., 2011; Marques and Pereira, 2011), Global Reaction Route Mapping or GRRM (Ohno and Maeda, 2006, 2019), Automated Mechanisms and Kinetics or AutoMeKin (Martínez-Núñez, 2015a,b; Martínez-Núñez, 2020), and Genetic Algorithm fitting or GAFit (Rodríguez-Fernández et al., 2017, 2020). Most of these computational programs are interfaced with well-known packages that perform electronic-structure calculations and, hence, allow for a direct assessment of the semi-empirical, density functional theory (DFT) or *ab initio* energy of the system during the optimization process. Another relevant methodology to explore low-energy landscapes is the parallel-tempering Monte Carlo technique, which has been also applied in the calculation of thermodynamic properties.

Global geometry optimization studies are, now, being extended to systems of increasing complexity. In particular, global optimization algorithms have been applied to a great diversity of chemical systems, including atomic and molecular clusters as well as colloidal aggregates and biomolecules. Nonetheless, optimization work needs, in general, a large number of computational resources and, hence, improvements in algorithms to relieve the burden. Major challenges are concerned with the treatment of systems with increasing size and incorporating higher levels of theory in the molecular model. Also, multi-component aggregates pose an important combinatorial problem and require novel optimization strategies. Although the use of state-of-the-art spectroscopic techniques to probe the structure of clusters has allowed for close collaborative work involving computational and experimental achievements, there is still room for greater improvement in this effort. In particular, comparisons between theoretical and experimental spectroscopic data will benefit from significant improvements in algorithms devoted for the spectral assignment.

Pursuing those purposes, we believe the collection of papers for the present Research Topic illustrates the broad scope of computational strategies for global optimization applications in chemistry. All contributions describe optimization strategies for a great diversity of chemical systems.

Basin-hopping (BH) is able to generate a coarse-grained mapping of a potential energy surface (PES) in terms of local minima, which can then be used to gain insights into molecular dynamics and thermodynamic properties as pointed out by Zhou et al. in their contribution. These authors also show how unsupervised machine learning tools can be employed to enhance BH searches, which result in more efficient identification of local minima and transition states connecting them.

Jana et al. employ a particle swarm optimization (PSO) method to search for small C_n_ clusters. PSO is another useful algorithm for a stochastic search in multidimensional space. The method has proven efficient in hard optimization problems compared with traditional methods.

Hernández-Rojas and Calvo also employ BH method, this time to predict low-energy structures of adamantane clusters by using both coarse-grained and atomistic potential models. Although coarse-grained models are appealing for the complex clusters that are studied, the comparison with atomistic potentials shows that some relevant structural details are not captured by the former.

As for seeking conformational minima of flexible acyclic molecules, Ferro-Costas and Fernández-Ramos propose an algorithm that combines a systematic variation of torsion angles with a Monte Carlo search. This methodology has been applied to calculate multi-structural partition functions of several alcohols ranging from n-propanol to n-heptanol and was also tested with the amino acid L-serine.

Panadés-Barrueta et al. put forward a fully automated method to generate highly-accurate semiempirical potential energy surfaces. They use global optimization techniques and automated PES sampling algorithms to refine specific reaction parameters of semi-empirical Hamiltonians, which can be subsequently employed in quantum dynamics studies.

In turn, Wang et al. carry out a microsolvation study of Na^+^ with water by applying a genetic algorithm combined with density functional theory to obtain low-energy structures of the clusters. Also, a new genetic algorithm is proposed by Silva et al. for the prediction of structures of nanoparticles. This work explores the efficacy of new evolutionary operators to treat Lennard-Jones and carbon clusters.

Khatun et al. develop a global optimizer which grows the cluster by adding atoms one by one. The method is tested by studying transition-metal clusters and binary and ternary nanoalloys of such elements.

Cova and Pais review and discuss deep learning strategies for optimizing the prediction of chemical patterns, which includes accelerated literature searches, analysis and prediction of physical and quantum chemical properties, transition states, chemical structures, chemical reactions, and also new catalysts and drug candidates.

## Author Contributions

All authors listed have made a substantial, direct and intellectual contribution to the work, and approved it for publication.

### Conflict of Interest

The authors declare that the research was conducted in the absence of any commercial or financial relationships that could be construed as a potential conflict of interest.

## References

[B1] Alexandrova A. N. Boldyrev I. (2005). Search for the Li${}_{\text{n}}^{0/+1/-1}$ (*n* = 5–7) lowest-energy structures using the *ab Initio* gradient embedded genetic algorithm (GEGA). Elucidation of the chemical bonding in the lithium clusters. J. Chem. Theory Comput. 1, 566–580. 10.1021/ct050093g 26641677

[B2] Dieterich J. M. Hartke B. (2017). OGOLEM: Framework for GA-Based Global Optimization. Available online at: https://www.ogolem.org/ (accessed March, 2020).

[B3] Hartke B. (1993). Global geometry optimization of clusters using genetic algorithms. J. Phys. Chem. 97, 9973–9976. 10.1021/j100141a013

[B4] Johnston R. L. (2003). Evolving better nanoparticles: genetic algorithms for optimising cluster geometries. Dalton Trans. 4193 10.1039/B305686D

[B5] Llanio-Trujillo J. L. Marques J. M. C. Pereira F. B. (2011). An evolutionary algorithm for the global optimization of molecular clusters: application to water, benzene, and benzene cation. J. Phys. Chem. A 115, 2130–2138. 10.1021/jp1117695 21370815

[B6] Marques J. M. C. Pereira F. B. (2011). EA_MOL: Evolutionary Algorithm for the Global Minimum Search of Molecular Clusters. Available online at: https://apps.uc.pt/mypage/faculty/qtmarque/en/software/ (accessed February, 2020).

[B7] Martínez-Núñez E. (2015a). An automated method to find transition states using chemical dynamics simulations. J. Comput. Chem. 36, 222–234. 10.1002/jcc.23790 25413470

[B8] Martínez-Núñez E. (2015b). An automated transition state search using classical trajectories initialized at multiple minima. Phys. Chem. Chem. Phys. 17:14912. 10.1039/C5CP02175H 25982874

[B9] Martínez-Núñez E. Barnes G. L. Glowacki D. R. Kopec S. Pelaez-Ruiz D. Rodriguez A. (2020). AutoMeKin: Automated Mechanisms and Kinetics. Available online at: https://rxnkin.usc.es/index.php/AutoMeKin (accessed January, 2020).

[B10] Ohno K. Maeda S. (2006). Global reaction route mapping on potential energy surfaces of formaldehyde, formic acid, and their metal substituted analogues. J. Phys. Chem. A 110, 8933–8941. 10.1021/jp061149l 16836457

[B11] Ohno K. Maeda S. (2019). Distribution of GRRM. Available online at: https://iqce.jp/GRRM/index_e.shtml (accessed February, 2020).

[B12] Rodríguez-Fernández R. Pereira F. B. Marques J. M. C. Martínez-Núñez E. Vázquez S. A (2017). GAFit: a general-purpose, user-friendly program for fitting potential energy surfaces based on genetic algorithms. Comput. Phys. Commun. 217:89 10.1016/j.cpc.2017.02.008

[B13] Rodríguez-Fernández R. Pereira F. P. Marques J. M. C. Vázquez-Rodríguez S. Martinez-Nunez E. (2020). GAFit, version 1.6. Available online at: https://rxnkin.usc.es/index.php/GAFit (accessed January, 2020).

[B14] Shayeghi A. Götz D. Davis J. B. A. Schäfer R. Johnston R. L. (2015). Pool-BCGA: a parallelised generation-free genetic algorithm for the *ab initio* global optimisation of nanoalloy clusters. Phys. Chem. Chem. Phys. 17, 2104–2112. 10.1039/C4CP04323E 25482360

[B15] Wales D. J. (2010). GMIN: A Program for Finding Global Minima and Calculating Thermodynamic Properties From Basin-Sampling. Available online at: http://www-wales.ch.cam.ac.uk/GMIN/ (accessed January, 2020).

[B16] Wales D. J. Scheraga H. A. (1999). Global optimization of clusters, crystals, and biomolecules. Science 285, 1368–1372. 10.1126/science.285.5432.1368 10464088

